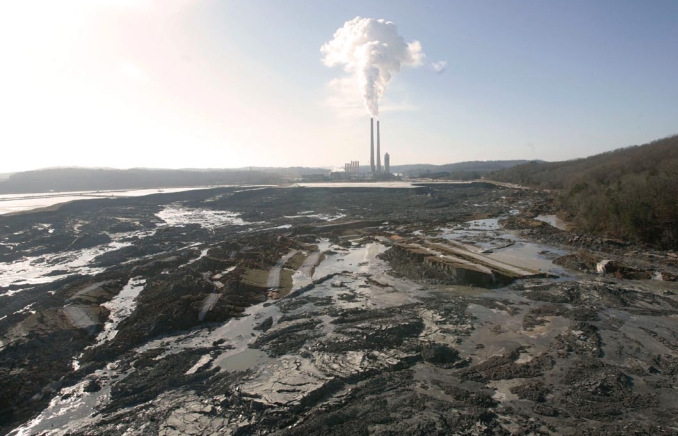# Balancing Act: Creating the Right Regulation for Coal Combustion Waste

**DOI:** 10.1289/ehp.117-a498

**Published:** 2009-11

**Authors:** John Manuel

**Affiliations:** **John Manuel** of Durham, North Carolina, is a regular contributor to *EHP* and the author of *The Natural Traveler Along North Carolina’s Coast* and *The Canoeist*

With the 22 December 2008 collapse of a Tennessee Valley Authority (TVA) ash pond in Kingston, Tennessee, and the arrival of the Obama administration the following month, the regulatory ground is shifting in regards to coal combustion waste (CCW), the millions of tons of waste left over each year from burning coal for electricity. The U.S. Environmental Protection Agency (EPA) is pursuing a host of initiatives that could directly or indirectly affect the disposition of CCW. States, too, are revisiting their regulations. The goal of these initiatives is to ensure public health; the challenge is to do so without compromising a recycling industry that the American Coal Ash Association (ACAA) says keeps about 56 million tons of CCW per year out of the landfilled waste stream.

## Current Disposition of CCW

CCW is created in the process of burning coal to generate steam in electric power plants. The approximately 440 coal-fired power plants in the United States are located primarily in the East, but can be found in virtually every state. These plants generated roughly 131 million tons of CCW in 2007, according to the ACAA.

This waste takes the form of fly ash, bottom ash, boiler slag, and flue gas desulfurization (FGD) materials. Fly ash is captured in the chimneys of coal-fired power plants, while the heavier bottom ash and boiler slag are collected from the bottom of the furnace. FGD materials are produced by emission control systems in which an alkaline agent, primarily limestone, is sprayed into the smoke stream to remove acid gases and some heavy metals. The resulting FGD sludge is dried before reuse.

CCW contains varying levels of the same potentially toxic elements that are found in coal. These include arsenic, chromium, lead, cadmium, selenium, and mercury. Numerous studies, such as work published in the May 2006 issue of *EHP* by William Alexander Hopkins and colleagues, have documented adverse effects, including developmental and behavioral abnormalities, in fish and amphibians exposed to CCW constituents that have been released to aquatic ecosystems.

About 43% of the CCW produced each year is recycled, primarily as a replacement for portland cement in concrete mix and, in the case of FGD gypsum, as a replacement for virgin gypsum in wallboard. These uses conserve landfill space, reduce the demand for virgin materials, and reduce carbon dioxide emissions caused by the extraction of virgin materials and the manufacture of finished products. A recent analysis by the Electric Power Research Institute (EPRI) and the Recycled Materials Resource Center at the University of New Hampshire suggests that such “beneficial uses” saved about 160 trillion BTUs of energy, 11 million tons of carbon dioxide equivalent, and 32 billion gallons of water in 2007. [For more information about construction-related uses of CCW, see “Trash or Treasure? Putting Coal Combustion Waste to Work,” p. A490 this issue.]

CCW that is not recycled typically is stored on-site in landfills or stored long-term in ponds (wet storage). Ash ponds, which are often built behind earthen dams or retaining walls, can pose a direct threat to human health and the environment should their bounds fail. This was what happened at TVA’s Kingston Fossil Plant in a spill that spread coal ash over 300 acres and damaged a dozen homes.

## Questioning the Safety of CCW Storage

Following that event, the EPA requested information from electric utilities around the nation to assess the structural integrity of their ash ponds. The survey responses, published on the Special Wastes section of the EPA website, identified 584 impoundments, 49 of which were considered to have “high hazard potential” according to Federal Emergency Management Agency classifications for dams—that is, the volume and/or siting of the dam make it “probable” that human death and possibly major environmental damage will occur if the dam were to fail (this rating does not, however, speak to the potential toxicity of the dammed material or to the likelihood the dam will fail). Another 60 impoundments were rated as having “significant hazard potential.” In this case, the concern is primarily economic and environmental loss; human deaths are not expected.

However, says John Suttles, a senior attorney at the Southern Environmental Law Center (SELC), “Many utilities in the Southeast U.S. refused to provide complete data such as size and volume of waste ponds, citing confidential business information. Most other utilities divulged this information and made no such claims of confidentiality.”

The EPA has followed up its survey with onsite assessments of all units having a potential rating of “high” or “significant” hazard. Reports on the first 17 units were published in September 2009; the EPA rated the structural integrity of 7 of these impoundments as “satisfactory,” 9 as “fair,” and 1 as “poor.” All assessments are to be completed by the end of the year.

TVA, for its part, reported to the EPA that its Kingston Fossil Plant had been rated as having “low hazard potential” at its last assessment in October 2008. However, cleanup of the site, currently under way, will cost an estimated $1 billion.

A preliminary assessment of the ecologic consequences of the Kingston spill, published in the 1 August 2009 issue of *Environmental Science & Technology* by a consortium of Duke University scientists and engineers, identified elevated levels of arsenic, selenium, lithium, and boron in a tributary of the Emory River that had been dammed by the spilled ash. Concentrations of dissolved arsenic in this pond reached 86 μg/L, compared with upstream water concentrations of 0.1–0.4 μg/L. Mercury concentrations of 92–130 μg/kg in sediments downstream of the spill were almost as high as those in the CCW itself.

Concentrations of these elements were significantly lower at sampling sites downstream on the Emory and Clinch Rivers but still were above background concentrations. Levels of radioactivity were slightly higher than those in ambient soil at the site, although according to the U.S. Geological Survey, CCW radioactivity generally is comparable to that of natural sources including some shales and granitic and phosphate rocks.

Aside from the direct danger of failing dams, CCW disposal sites can pose a potential threat to human health and the environment by virtue of the leaching of toxic constituents into groundwater when appropriate protective measures are not in place. These chemicals may then be conveyed to nearby drinking water wells and surface waters. A 2007 EPA study, *Coal Combustion Waste Damage Assessments*, identified 24 cases of proven damage and 43 cases of potential damage to ground or surface water adjacent to CCW disposal sites. Damage was “proven” when exceedances of health-based standards were documented in water far enough from the disposal site “to indicate that hazardous constituents have migrated to the extent that they could cause human health concerns,” according to the report.

The year before, the EPA commissioned Research Triangle Institute to perform an analysis of the health risks of landfills and storage ponds containing CCW. The authors assessed soil and aquifer data within a 5-km radius of each of 181 selected coal-fired power plants. In a draft report titled *Human and Ecological Risk Assessment of Coal Combustion Wastes*, the authors modeled elevated cancer risk from arsenic for people who drank water from wells near unlined or clay-lined surface impoundments. They also modeled elevated noncancer risks from molybdenum, boron, lead, cadmium, and cobalt. The report has remained unpublished in final form for two years, but EPA spokeswoman Latisha Petteway says it has now been peer reviewed and that a final version is scheduled for publication in December 2009.

Contaminated water is not the only problem associated with CCW storage. Fine ash particles also can be transported by wind and may be inhaled. Moreover, some scientists and advocacy groups believe certain “beneficial uses” of CCW, for instance as fill in unlined mines, may pose these same water and air quality threats.

## Regulatory Options

As far back as 1980, the EPA has debated designating CCW as a hazardous waste under Subtitle C of the Resource Conservation and Recovery Act (RCRA). A Subtitle C designation would mean CCW must be managed as a hazardous waste “from cradle to grave.” Under Subtitle C, federal-level regulations govern the generation, transportation, and treatment, storage, and disposal of hazardous wastes. RCRA’s Subtitle D, in contrast, addresses nonhazardous solid wastes including household garbage, sludge from industrial and municipal wastewater plants and pollution control facilities, and certain hazardous wastes that are exempt from Subtitle C regulations (such as household hazardous waste). State and local governments are responsible for regulating Subtitle D wastes, with guidance from the EPA.

But in two separate regulatory determinations, the EPA determined CCW should not be regulated under Subtitle C, nor under Subtitle D, for that matter. Instead, regulation has been left to the states, which have generally included disposed CCW under municipal solid waste or industrial waste regulations, if they regulate CCW at all (36 states have permit programs for CCW landfills).

The EPA is now pushing forward on a number of policy fronts, including stricter regulation of CCW disposal sites, as well as another foray into possibly classifying CCW as a hazardous waste under RCRA. Matthew Hale, director of the EPA Office of Resource Conservation and Recovery, says, “EPA is considering a range of options, including regulation of coal combustion under Subtitle D authority, under Subtitle C authority, and combined options that involve a mixture of Subtitle C and Subtitle D authorities. EPA . . . will propose one option as the preferred approach, but will take comment on a full range of options.”

RCRA offers two main avenues by which a material can be classified as a hazardous waste under Subtitle C. The first avenue is to specifically list CCW in the act, which assumes that all CCW is of a uniform nature as far as toxic constituents go. RCRA also has provisions that define a listed waste as hazardous but allow certain specified uses of the waste that are unlikely to pose a threat. The second is through a set of technical analyses known as the toxicity characteristic leaching procedure. If, during testing, the material leaches more than a specified concentration of certain constituents, it is deemed “hazardous by characteristic.”

Very little CCW sampled has failed the toxicity characteristic leaching procedure. However, in an 8 September 2008 presentation at the Global Waste Management Symposium, EPA scientist Susan Thornloe and colleagues reported that efforts are currently under way to incorporate more reliable CCW leach tests into the EPA’s SW-846, a compendium of accepted test methods for evaluating solid waste. Past CCW testing, they noted, has not always considered field conditions known to influence leaching. Furthermore, assessments have sometimes considered initial but not final site conditions, although changes at a site over time could change the propensity of a material to leach.

## Hazardous or Not?

The distinction ultimately chosen for CCW will have tremendous implications for recycling and disposal. Few would disagree that some of the constituents in CCW can be toxic under certain conditions (for instance, in drinking water). At the same time, many assert that classification of all CCW as “hazardous” under Subtitle C is unjustified and would severely constrain, if not altogether end, recycling of the material. “The marketplace would not choose to use something designated hazardous waste when they have other options,” says Tom Adams, ACAA executive director.

If classified as hazardous, utility companies would be left with more than 50 million additional tons of CCW to dispose of each year under strict regulations. This waste would have to be transported, in some cases long distances, to landfills specifically designed and permitted to handle hazardous wastes. EPRI, using a cost estimate prepared for the Utilities Solid Waste Action Group of the Edison Electric Institute, modeled a tenfold increase in disposal costs, which are currently around $1 billion annually. In addition, existing facilities might have to be closed or, if possible, retrofitted.

For this reason, electric utility and coal industry officials are strongly opposed to Subtitle C classification. So, too, is the Association of State and Territorial Solid Waste Management Officials (ASTSWMO), which voiced its opinion in an April 2009 letter to Hale. “Coal combustion byproducts rarely if ever fail the criteria by which materials are determined to be hazardous waste,” the authors wrote. “To artificially classify them as hazardous waste will needlessly limit the management options for both [CCW] and other waste classified as hazardous, which will be competing with [CCW] for limited hazardous waste disposal capacity, while not providing any greater degree of environmental protection. . . . The prospect of adding a significant new waste stream to be managed by severely underfunded State hazardous waste programs is unconscionable unless a significant amount of new sustained funding is included.”

ASTSWMO is also opposed to a hybrid Subtitle C and D designation in which disposed material is classified as hazardous whereas recycled material is classified as solid waste. “The uncertainty that a presumed hazardous waste material could be deemed hazardous as a result of a determination that a generator failed to follow Subtitle D requirements will create too much uncertainty and liability concerns for the beneficial user,” the group wrote in its letter. Instead, the organization encourages EPA to classify all CCW under Subtitle D, with implementation left to the states and enforcement by the federal government under RCRA.

Environmental groups do not want to end all genuinely beneficial uses of CCW, but they do want stiffer requirements for disposed waste than are called for under Subtitle D, and they also have serious concerns about unconsolidated land-based uses such as structural fills and agricultural applications, says Chandra Taylor, a senior attorney at the SELC. “There’s no question that CCW has hazardous constituents, and the EPA should promulgate rules in consideration of that fact,” says Taylor. “Certainly, we want at least minimum federal standards for disposal—a dual liner for landfills, a leachate collection system, testing for contaminants in the groundwater supplies, and phaseout of wet storage.”

Industry spokespeople say the EPA is indeed likely to call for a phaseout of wet storage. TVA has already announced that it will phase out wet storage within eight years at an estimated cost of $1.5–2.0 billion to pay for new ash-handling equipment and storage facilities. But industry groups remain opposed to any mandatory closure of existing ponds.

“Mandatory pond closure is not necessary to ensure safe management of [CCW],” says James Roewer, executive director of the Utilities Solid Waste Action Group. “The ‘nonhazardous’ regulations we are advocating would allow those ponds that are managing [CCW] without adverse environmental impact to continue to do so. Why should a disposal facility that is safely managing [CCW] be forced to close? The costs of conversion of ponds to landfills are huge—as much as $39 billion across the industry.”

“Whatever EPA decides, it’s going to require a lot of new landfill space,” Adams says. “And new space is hard to identify and hard to permit.”

## A State Perspective

Irrespective of federal classification, states are moving toward stricter regulation of CCW used in various applications, most notably structural fills. This practice, a popular use of CCW in many states, involves depositing the waste at the core of earthen fills that are subsequently built upon as recreational, commercial, or industrial sites. The key to the environmental safety of these structural fills is preventing water from leaching through the core and mobilizing toxic constituents of the CCW.

Most states do not require monitoring of groundwater around most structural fills, so there is little information on the potential for leaching from these facilities. But several sites with environmental concerns have been cited, and at least one case of potentially serious concern has been detected. In Chesapeake, Virginia, heavy metals have been detected in the groundwater surrounding a golf course built on 1.5 million tons of fly ash. Residents are suing Dominion Virginia Power Company to have the ash removed and public water and sewer brought to their neighborhoods. The EPA continues its study and monitoring in this case; the extent of the damage and a definitive link to the golf course have not been established, according to Petteway.

Virginia’s Department of Environmental Quality has formed an advisory committee to look at strengthening the regulations for structural fills using CCW. “There are going to be some new requirements to limit the permeability of fills,” says W. Lee Daniels, a professor of crop and soil environmental sciences at Virginia Tech and a member of the committee. “New regulations would not allow a fill to be built in the 100-year floodplain.”

## The Impact of Mercury Regulation

Other federal-level policy initiatives may impact the future of CCW by imposing pollution control technologies that affect the quality of fly ash. The EPA is currently in the process of developing standards under the Clean Air Act for reduction of mercury and other pollutants emitted by oil- and coal-fired power plants. The EPA’s new rules would supplant a plan passed by the Bush administration in 2006 that would have allowed power companies to avoid controls by purchasing emission “credits” from power plants in other parts of the country. A trading system can make sense for greenhouse gases that are widely dispersed, but experts agree it is not appropriate for mercury, which deposits locally.

The EPA is currently acquiring data from utilities that have implemented various control technologies at their power plants to determine what levels of reduction are feasible for mercury and other toxics included in the list of 187 hazardous air pollutants the agency is required to regulate under the Clean Air Act. The EPA has already found that coal-burning power plants emit 67 of these pollutants, notes the SELC’s Suttles. Some technologies, such as activated carbon injection, have proven highly effective in reducing mercury emission.

However, says Suttles, a great deal of pollution that is removed from power plant emissions—and thus goes into CCW—ends up being emitted into the air anyway from cement plants when these plants use CCW as a feedstock. This was the thinking behind a New York State announcement on 13 October 2009 that cement manufacturer Lafarge North America will no longer be allowed to use fly ash in its Ravena cement plant. The announcement follows the measurement of elevated levels of mercury in the soil and wildlife surrounding the plant.

Moreover, depending on how activated carbon injection is incorporated, it can alter the air-entrainment characteristics of fly ash, which are important to helping concrete resist the stress of freeze/thaw cycles. These alterations render the fly ash potentially less desirable for use as a concrete feedstock.

The latter “may not be a showstopper,” says Ken Ladwig, EPRI senior program manager. EPRI and other groups are researching technologies for collecting fly ash separately from the injected carbon and for treating fly ash to minimize the effects of the injected carbon on concrete. “Any of these are going to raise the cost [of using fly ash in concrete],” says Ladwig. But because incorporation as a concrete component is the largest recycling use of CCW at more than 14 million tons per year, any practice that reduces this option would also increase CCW disposal costs and amounts.

## Balance

EPA has yet to finalize any of its proposed rules, so it is too early to say what requirements will be placed on individual power plants and what technologies the utilities will use to achieve those requirements.

Suttles, a staunch advocate of strict controls on power plant emissions, is cognizant of the impacts that new regulations may have on disposal costs and recycling of CCW. But he does not believe such impacts are justification for compromising public health and safety.

“We are starting to see the true cost of using coal as an energy source,” he says. “Yes, we may be taking pollution that would have gone into the air and creating a land disposal problem, [but] our position is that you have to account for all these risks. The result is that using coal is not cheap. We need to be pushing energy efficiency and renewable energy.”

## Figures and Tables

**Figure:**
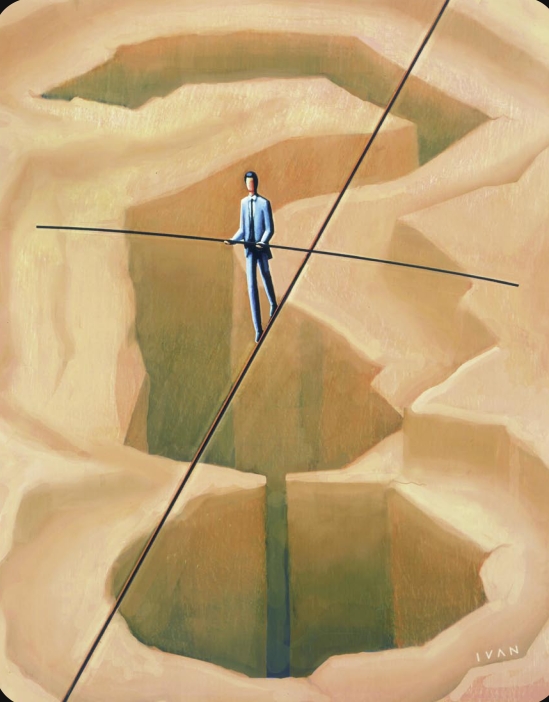


**Figure:**
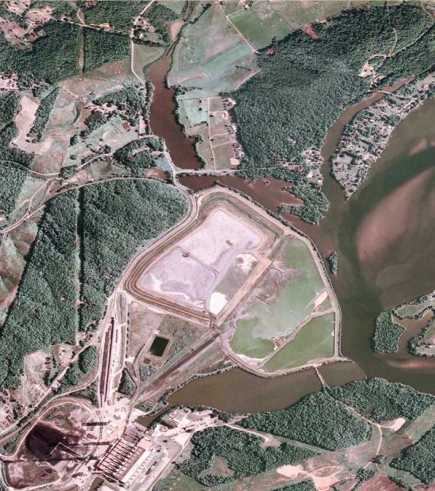
Above: Aerial photos of the Kingston Fossil Plant showing the storage pond in June 2007 (left) and 8 days after a dam failed on 22 December 2008 (right). Below: More than 5 million cubic yards of waste was released into the Emory River during the Kingston spill. A preliminary assessment published 8 months later revealed elevated levels of arsenic and mercury in the river water and sediment.

**Figure:**
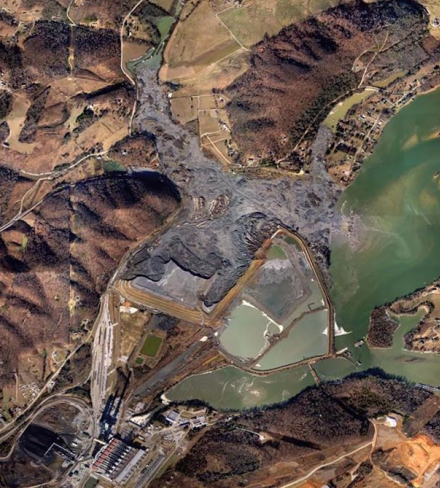


**Figure:**